# Conformational Essentials Responsible for Neurotoxicity of Aβ42 Aggregates Revealed by Antibodies against Oligomeric Aβ42

**DOI:** 10.3390/molecules27196751

**Published:** 2022-10-10

**Authors:** Chuli Song, Tianyu Zhang, Yingjiu Zhang

**Affiliations:** 1 Key Laboratory for Molecular Enzymology and Engineering of the Ministry of Education, Jilin University, Changchun 130012, China; 2 School of Life Science, Jilin University, Changchun 130012, China

**Keywords:** Alzheimer’s disease (AD), amyloid β-protein (Aβ), neurotoxicity, antibody, aggregation

## Abstract

Soluble aggregation of amyloid β-peptide 1-42 (Aβ42) and deposition of Aβ42 aggregates are the initial pathological hallmarks of Alzheimer’s disease (AD). The bipolar nature of Aβ42 molecule results in its ability to assemble into distinct oligomers and higher aggregates, which may drive some of the phenotypic heterogeneity observed in AD. Agents targeting Aβ42 or its aggregates, such as anti-Aβ42 antibodies, can inhibit the aggregation of Aβ42 and toxicity of Aβ42 aggregates to neural cells to a certain extent. However, the epitope specificity of an antibody affects its binding affinity for different Aβ42 species. Different antibodies target different sites on Aβ42 and thus elicit different neuroprotective or cytoprotective effects. In the present review, we summarize significant information reflected by anti-Aβ42 antibodies in different immunotherapies and propose an overview of the structure (conformation)−toxicity relationship of Aβ42 aggregates. This review aimed to provide a reference for the directional design of antibodies against the most pathogenic conformation of Aβ42 aggregates.

## 1. Aβ42 Oligomers Are the Most Pathogenic Aβ Species

**Figure 1:**
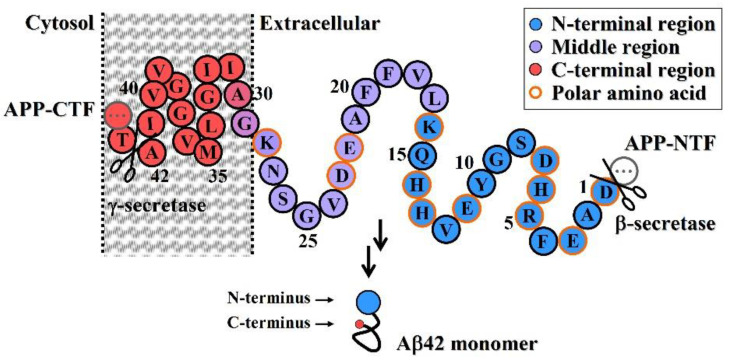
Generation of Aβ from the processing of APP by β- and γ-secretases. On the transmembrane substrate APP molecule, β-secretase has only one cleavage site, while γ-secretase has multiple cleavage sites; therefore, the resulting Aβ peptides have the same N-terminus but different C-termini, where Aβ42 is the major secretory product.

The aggregation and deposition of Aβ42 are typical events in Alzheimer’s disease (AD) pathogenesis. AD is characterized by a series of adverse biological effects triggered by Aβ42 aggregation and deposition. Amyloid β-peptides are a class of small isoforms that originate from sequential proteolytic cleavage of the amyloid precursor protein (APP) located on the membrane of human brain cells by β- and γ-secretases. Due to the diverse cleavage of APP by γ-secretase in the intramembrane region, β-amyloid is composed of 38–43 amino acids (Aβ38 to Aβ43), of which Aβ42 is the most abundant product (Figure 1). Aβ42 is a physiologically relevant peptide; in healthy individuals, Aβ42 is present in small quantities as a soluble monomer. However, Aβ42 is an amphiphilic molecule with a hydrophilic N-terminal region and a hydrophobic C-terminal region, in which the C-terminal 12-amino acid sequence (29GAIIGLMVGGVVIA42) (Figure 1) of the transmembrane region of APP has strong hydrophobicity. 

Therefore, Aβ42 is a hydrophobic molecule according to its grand average hydropathicity (GRAVY, 0.205) [1], although it contains six negatively charged residues (Asp + Glu), three positively charged residues (Lys + Arg), and three His residues (Figure 1). Under physiological conditions, its C-terminal hydrophobic region forms a tight intramolecular hydrophobic interaction through folding of the C-terminal main chain and exposes the hydrophilic N-terminal region. Its native conformation (folded) enables it to exist stably as a monomer in vivo and in vitro without self-aggregation. Secreted Aβ42 is likely to play important physiological roles in organisms, including trophic activity [2,3].

**Figure 2:**
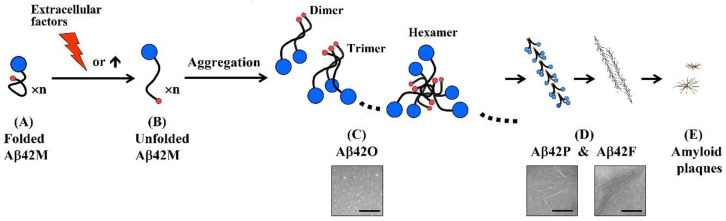
Unfolded (or misfolded) Aβ42 monomers are prone to self-aggregation to form different aggregate species. Abbreviations: Aβ42M/O/P/F, Aβ42 monomers, oligomers, protofibriles, and fibrils. Blue and red circle(s) in (**A**–**D**): N-terminal and C-terminal regions, respectively. E: schematic diagram of amyloid plaques. Electron microscopic image(s) in (**C**) and (**D**): Aβ42 oligomers, protofibriles, and fibrils. Scale bar = 80 nm.

However, certain factors, such as environmental changes, may induce a change in the conformation of Aβ42 from a natural compact state to an unfolded (or misfolded) state (Figure 2A,B), which can be considered degeneration of Aβ42. This unfolded Aβ42 is thermodynamically unstable, and the exposed C-terminal hydrophobic region is prone to self-aggregation to form Aβ42 aggregates driven by the hydrophobic interaction between the peptide chains (Figure 2B,C). As the concentration of Aβ42 increases, unfolded (or misfolded) Aβ42 is prone to self-aggregate into oligomers and further assemble into protofibrils, fibrils, and amyloid plaques (Figure 2D–E). The main components of Aβ aggregates in humans are Aβ42 and Aβ40. Because Aβ42 has two more hydrophobic amino acid residues (Ile-41 and Ala-42) at its C-terminus than Aβ40, Aβ42 is more hydrophobic than Aβ40 and more prone to aggregation than Aβ40, especially at a much lower concentration [4]. Furthermore, Aβ40 cannot form a stable S-oxidative radical cation due to the absence of Ile-41 and Ala-42 [4], so Aβ40 has a much lower neurotoxicity than Aβ42, which indicates the important role of Aβ42 in amyloidogenesis. Thus, Aβ42 is more directly linked to AD.

There is now a broad consensus that although different Aβ42 aggregates exhibit different adverse biological effects on neural cells, soluble Aβ42 oligomers (Aβ42Os), rather than Aβ42 fibrils (Aβ42Fs) or amyloid plaques, are regarded as the most pathogenic form of Aβ aggregates, which can cause more significant neurological damage in human and animal models of brain tissue and instigate major facets of AD neuropathology, including tau pathology, synapse deterioration or damage, neuronal loss, inflammation, and oxidative damage [5]. Considerable in vivo studies support the important role of Aβ42 oligomers in the pathogenesis of AD, including the induction of increased extracellular vesicle secretion [6], trigged pathophysiological signalings [7], and abnormally activated hippocampal microglial and astrocytic cells [8]. These reports all demonstrate that Aβ42 oligomers in AD brains shows a better correlation with memory impairment or cognitive decline than Aβ42 fibril or plaque accumulation. Therefore, the “Amyloid Cascade Hypothesis” [9,10], which postulates that the neurodegeneration in Alzheimer’s disease caused by abnormal accumulation of amyloid-β plaques in various areas of brain, has also developed into the “Amyloid-β Oligomer Hypothesis” [11], which postulates that the neurodegeneration in Alzheimer’s disease caused by abnormal accumulation of Aβ42 oligomers (Aβ42Os) in various areas of brain. Amyloid-β Oligomer Hypothesis highlights that the course of AD is positively correlated with the content of Aβ42 oligomers rather than Aβ42 plaque in the brain.

An increasing number of studies have demonstrated that in addition to directly destroying the integrity and permeability of neuronal cell membranes by forming membrane pores [12,13,14], extracellular Aβ42Os are mainly capable of binding to a variety of membrane receptors or membrane proteins on the surface of neural cells in a ligand-like manner, resulting in synaptic dysfunction and neurodegeneration through multiple abnormal alterations in the corresponding signaling pathways [5,14,15]. More than 20 putative receptors or membrane proteins have been reported to be associated with the neurotoxic activity of AβO oligomers, including the N-methyl-d-aspartate receptor [16,17,18], p75 neurotrophin receptor [19,20], prion-like protein [21,22], α-amino-3-hydroxy-5-methyl-4-isoxazole propionic acid receptor [18,23], and integrin receptors [24]. Notably, all these receptors or membrane proteins are often multi-subunit complexes. This suggests that the structural basis for the toxic activity of Aβ42Os is primarily the specific integrated conformation between Aβ42 chains within the Aβ42O unit rather than the conformation of individual Aβ42 chains.

## 2. A Specific Integrated Conformation Underlies the Neurotoxicity of Aβ42Os

Over 20 years ago, the first active AD vaccine AN1792 (human aggregated Aβ42) was verified to elicit a positive antibody response to Aβ42 and reduce plaque burden in transgenic mouse models of AD [25,26]. Several subsequent studies have shown that the N-terminal region of Aβ42 (Aβ1–15 or shorter Aβ) is the dominant B cell epitope that mainly induces a Th2-type humoral response (ratio of IgG1 to IgG2a: >1), while the C-terminal region of Aβ42 (Aβ16–30, Aβ19–33, and Aβ28–42) is the dominant T cell epitope that mainly causes a Th1-type cellular response (ratio of IgG1 to IgG2a: <1) [27,28]. Th2-type humoral responses have been shown to be safe and beneficial, as they do not cause adverse events. Antibodies induced by a variety of N-terminal fragments of Aβ42 (Aβ1–6, Aβ1–12, Aβ1–14, and Aβ1–15) [29,30,31,32] reduce the levels of Aβ1–42 oligomers, protofibrils, and plaque load and improve cognition in AD model mice, suggesting that the N-terminal region of theAβ42 chain is at least one of the structural sites responsible for the toxic activity of Aβ42 aggregates. 

**Figure 3:**
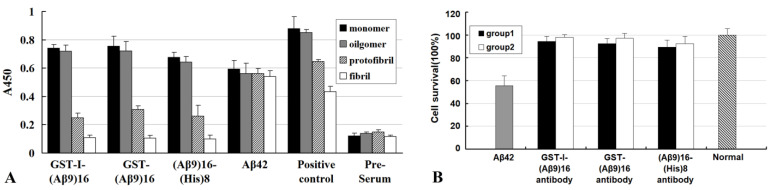
Immunoreactivity of two serum antibodies to all Aβ42 species (cited from [33]): (**A**) The pre-immune serum (pre-serum) used as the negative control; (**B**) Aβ42-specific antibody used as the positive control. Abbreviations: (Aβ9)16, sixteen tandem repeats of Aβ1-9 (Aβ9); GST, glutathione S-transferase; (His)8, eight-histidine tag (8 × His); I, immunoenhancing sequence that was composed of two helper T cell epitopes (pan HLA DR binding epitope (PADRE) and Tetanus toxin epitope (TT)). In (**B**), group 1 was a oligomeric Aβ42 cytotoxicity-neutralizing group, where Aβ42 oligomers was added into each well of 96-well plates and cells were cultured for 3 days, then were replenished with the purified (Aβ9)16-induced antibodies per well and continued to be cultured for another 3 days; group 2 was an oligomeric Aβ42 cytotoxicity-inhibiting group, where Aβ42 oligomers and the purified (Aβ9)-induced antibodies were added into each well of 96-well plates and cells were cultured for 6 days.

The structure-toxicity relationship of Aβ42 aggregates was further revealed by serum antibodies induced by Aβ1–9, Aβ1–28, and Aβ42 in our previous studies (Figure 3) [33,34]. It has been reported that the serum antibodies induced by (Aβ9)16 (16 tandem repeats of Aβ1–9) display a high immunoreactivity to Aβ42M and Aβ42O (*p* < 0.01, Aβ42M/Aβ42O and Aβ42P/Aβ42F) but a low immunoreactivity to Aβ42P (*p* < 0.05 compared with pre-serum) and no immunoreactivity to Aβ42 mature fibrils (*p* > 0.05 compared with pre-serum) (Figure 3A). In contrast, the serum antibodies induced by full-length Aβ42 do not show differences in immunoreactivity to any Aβ42 species (*p* > 0.05 between Aβ42 species groups) (Figure 3A). This indicates that antibodies induced by N-terminal fragments of Aβ42 (such as Aβ1–9) mainly recognize conformational epitope(s) integrated in Aβ42 aggregates in addition to linear epitope(s) on Aβ42 chain, whereas antibodies, induced by full-length Aβ42, similar to those induced by AN1792 [30], recognizes only linear epitope(s) on Aβ42 chain. Meanwhile, these reports [33,34] show that antibodies induced by N-terminal fragments of Aβ42 (such as Aβ1–9 or Aβ1–28), like those induced by full-length Aβ42, are able to neutralize and inhibit the cytotoxicity of Aβ42O, at least in vitro. Similar to Aβ42-induced antibodies, (Aβ9) 16-induced antibodies can significantly neutralize the cytotoxicity of Aβ42 aggregates and restore cell viability to approximately 90% of normal viability in group 1 (cytotoxicity-neutralizing group) or can inhibit the cytotoxicity of Aβ42 aggregates and remain cell viability at more than 95% in group 2 (cytotoxicity-inhibiting group) compared with in the toxic control group (incubated only with Aβ42 oligomers for six days) (70%) (Figure 3B). Similar results have also been reported for Aβ1–6-induced and Aβ1–12-induced antibodies [35,36]. Antibodies targeting the N-terminal region of Aβ42 have a high binding specificity for Aβ42M and/or Aβ42O and can effectively block Aβ42O-induced neurotoxicity in vivo and/or in vitro. Several other monoclonal antibodies have also been identified with similar specificity for recognizing N-terminal epitopes on Aβ molecules, such as targeting Aβ1-8 [37], Aβ2-8 [38], and Aβ1-7 [39] epitopes.

Through a comparative analysis of the correlation of the above immunogens, induced immune responses, and antibody species, it was found that using the N-terminal fragments of Aβ42 as an immunogen, the antibodies induced by them have a high specificity for oligomeric Aβ42, but a low specificity for fibrils, which can effectively reduce the cytotoxicity of oligomerized Aβ42. Therefore, the following conclusions can be drawn:

(1) An antibody molecule usually recognizes only the exposed portions of an antigenic unit. The high binding specificity of antibodies induced by various N-terminal fragments of Aβ42 for Aβ42O demonstrates that the proportion of surface-located N-terminal regions is much higher in Aβ42O than in Aβ42P or Aβ42F (Figure 2C–E). In protofibrils and fibrils, the N-terminal region of Aβ42 is most likely distributed on the surface and inside in a closely juxtaposed manner, as shown in Figure 2D. Thus, the solubility of Aβ42P and Aβ42F is much lower than that of Aβ42O because of the hydrophilicity of the N-terminal region and hydrophobicity of the C-terminal region of the Aβ42 chain.
(2) The integrated conformation of Aβ42 aggregate species is closely related to its toxic activity; therefore, the binding specificity of an antibody against different Aβ42 aggregate species largely determines its efficacy in blocking or neutralizing the neurotoxicity of Aβ42 aggregates.
(3) Neuroprotective efficacy of antibodies induced by various N-terminal fragments of Aβ42 reveals that the exposed N-terminal region, approximately the first 16 amino acids of Aβ42 (DAEFRHDSGYEVHHQK) (Figure 1), appears to be the major structural element constituting the effector site responsible for Aβ42O neurotoxicity [33,34,35,36]. It is speculated that the N-terminally integrated structures of Aβ42O appear to be directly involved in binding to the membrane receptors and/or membrane structures of neural cells, thereby acting as alternative ligands to competitively or non-competitively disrupt some normal signaling pathways.
(4) The C-terminal and central regions of an Aβ42 chain and their interactions indirectly affect the N-terminal integration structure, so they are also structural factors affecting Aβ42O toxicity. Any factor that disrupts the central and C-terminal regions of the Aβ42 chain may indirectly affect the integrated conformation of the N-terminus of Aβ42O, thereby affecting the toxicity of Aβ42O.

## 3. Structure−Toxicity Relationships of Aβ42 Aggregates Revealed by Passive Immunization

The advantage of passive anti-Aβ immunotherapy is that the dose and specificity of the antibodies are controllable. Many in vitro and in vivo studies have shown that antibodies against Aβ42 can interfere with Aβ42 aggregation, block the toxicity of Aβ42 aggregates and reduce the amount of Aβ42 in the brain. The specificity of an antibody against different Aβ aggregates reflects the function or efficacy of the antibody to block or neutralize the neurotoxicity of Aβ aggregates. The mechanisms of action of anti-Aβ42 antibodies include inhibition of Aβ42 aggregation, induction of disaggregation and allostery of small Aβ42 aggregates, neutralization of Aβ42 aggregate neurotoxicity, and reduction of the Aβ42 burden in the brain.

During the past 20 years, a variety of anti-Aβ monoclonal antibodies (mAbs) [40,41], including bapineuzumab [42,43], ponezumab [44,45], solanezumab [46,47,48], gantenerumab [49,50], crenezumab [48,51,52,53], aducanumab [54,55], and BAN-2401 [56], have already entered clinical trials. Unfortunately, these monoclonal antibodies have rarely shown efficacy in clinical trials. On 7 June 2021, aducanumab became the first FDA-approved new drug for AD treatment in 18 years, but a new randomized controlled trial is still required to verify the clinical effect of aducanumab. Aducanumab is a human IgG1 monoclonal antibody that interacts with the N-terminal region of Aβ42. The humanized murine monoclonal antibody, BAN-2401, is the only passive immunization antibody used in phase III clinical trials. It has been shown to be highly safe and well tolerated in phases I and II clinical trials, but its therapeutic effect is unclear [40,41,56,57].

In addition, donanemab, another humanized monoclonal antibody against the N-truncated pyroglutamate-modified Aβ peptide at position 3 (AβpE3) [58], has recently gained attention, as a specialty antibody AβpE3 is a form of modified Aβ that is located solely within cerebral Aβ plaques and is not found in body fluids (cerebrospinal fluid or plasma); thus, donanemab has been claimed to react abundantly with Aβ plaques [59]. Donanemab appears to have demonstrated significant plaque clearance efficacy and was recently assessed in a phase 2 trial for its efficacy and safety for the treatment of early AD [58,60]. 

Anti-oligomeric Aβ42 single-chain variable fragment (scFv) antibodies are a promising class of antibodies for eliminating the neurotoxicity of Aβ42 aggregates. ScFv molecules are very small (approximately 1/6 of the whole antibody), but they contain the complete antigen-binding domains of an intact antibody, so it has a higher blood−brain barrier crossing and tissue penetration, while retaining the specificity of IgG to its antigen. Moreover, scFvs do not mediate deleterious inflammatory responses such as meningoencephalitis, cerebral microhemorrhages, or even death, due to the lack of the inflammatory Fc domain of mAbs. The above advantages of scFvs are favorable for their clinical application [61,62,63].

The toxicity sites of Aβ42 oligomers may be determined directly by the N-terminal sequence of Aβ42 and/or indirectly by the C-terminal and central regions of Aβ42. Designing scFvs targeting the N-terminal sequence of Aβ42 and inducing disaggregation or fragmentation of the N-terminal region is a promising research idea. Several anti-Aβ single-chain antibodies have been reported, and their efficacy in vitro and in vivo has been characterized [62]. Some of the scFvs targeting the N-terminal region of Aβ42 are shown in Table 1. These anti-oligomeric Aβ scFvs display high selectivity for toxic Aβ42O species, neutralize their neurotoxicity in vivo or in vitro and reduce the toxicity of preformed oligomeric Aβ42 toward target cells. In addition, some anti-oligomeric Aβ scFvs have been reported to have relatively significant permeability in in vitro blood−brain barrier models [71,72,73,74].

**Figure 4:**
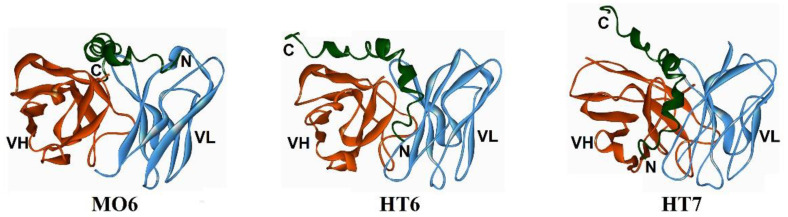
Overview of the conformations of three scFv antibodies (MO6, HT6, and HT7) with certain homology (linkers are not shown) [72,73,74].VH or VL: heavy or light chain variable domain; C or N: C- or N-terminus.

A comparative analysis of the similar binding models of our three scFvs (MO6, HT6, and HT7) to Aβ42O [72,73,74] revealed that the closer the scFv-bound site is to the N-terminus of Aβ42O (Figure 4), the larger the size of the corresponding scFv-bound Aβ42O target (Table 1) and vice versa. The relationship between the site on the Aβ42O unit targeted by a scFv antibody and the size of the Aβ42O target has implications for antibody function. For example, a scFv antibody with a binding site close to the N-terminus of Aβ42 would presumably have a relatively high ability to neutralize the toxicity of Aβ42O, based on the results that the N-terminally integrated structures of Aβ42O appear to directly result in the neurotoxicity of an Aβ42O unit. This has been demonstrated by the efficacies of the three scFvs (MO6, HT6, and HT7) in inhibiting Aβ42O toxicity by 3-(4,5)-dimethylthiahiazo (-z-y1)-3,5-di-phenytetrazoliumromide (MTT) assay [72,73,74]. The potency of these three scFv antibodies correlate with an aggregate set of in vitro activities, such as recognizing Aβ42 oligomers and fibrils in a consistent manner.

These studies suggest that anti-oligomeric Aβ42 scFvs may be an effective tool for AD diagnostics and therapeutics and may provide guidance for the development of improved antibody fragments against neurotoxic Aβ species associated with a specific neurodegenerative disease. Although the anti-Aβ scFv antibody test has not yet entered the clinical stage, it is speculated that anti-Aβ scFv antibodies have great development potential. In the near future, anti-Aβ scFv antibodies will open a window of hope for patients with AD.

## 4. Discussion and Prospects

**Figure 5:**
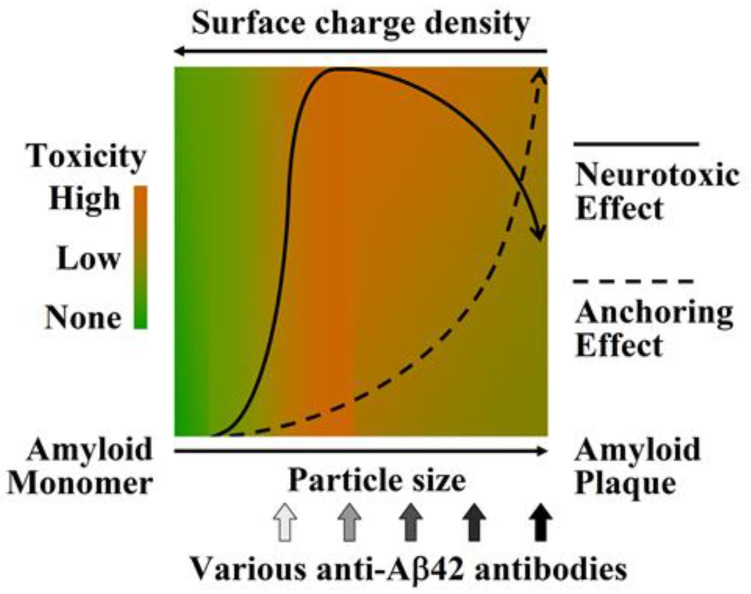
Schematic diagram of neurotoxicity of Abeta species. Neurotoxicity of Abeta is indicated by green (monomers), yellow-green (fibrils and amyloid plaques), or orange (oligomers) as none, low, or high, respectively.

Although fewer clinical benefits of antibodies have been reported thus far, the in vivo and in vitro binding properties and neuroprotective efficacy of antibodies, especially those targeting only the most pathogenic Aβ42O, provide us with many important clues to better understand the structure−toxicity relationship of various Aβ42 species. The specific and relatively stable three-dimensional conformations of proteins determine their biological function. However, for some small-molecule proteins such as Aβ42, their monomeric protein units usually do not have complex biological functions, but after they self-associate to form oligomeric structures, their oligomers usually gain novel functions that are beneficial or detrimental to living systems; however, Aβ42 is also detrimental. Aβ42 exhibits neurotoxic activity upon oligomerization. Furthermore, Aβ42Os continue to aggregate into large amyloid fibrils and plaques, in which the highly regular and non-branched structures correspond to super-secondary structures rather than tertiary structures, resulting in the insolubility of Aβ42F and plaques. Inevitably, the biological function of Aβ42F is significantly inferior to that of Aβ42Os, exhibiting only a constructive function similar to that of fibrous proteins. Thus, Aβ42Os are the most neurotoxic among all Aβ42 aggregate species, which also conforms to the rules concerning the structural and functional relevance of proteins. The properties and efficacies of various anti-Aβ42 antibodies are the most convincing validations for this. The correspondence between the properties and neurotoxicity of Aβ42 species is depicted in the schematic diagram in Figure 5.

According to the information reflected by the anti-Aβ42 antibodies involved in either active (i.e., immunization with Aβ42 or its fragments) or passive immunization (i.e., parenteral administration of anti-Aβ antibodies), the most likely implication of Aβ42O toxicity is that Aβ42Os act as alternative ligands or membrane-bound proteins, disrupting or destroying some normal signaling and membrane structures of neural cells, especially synaptic sites, as previously reported [75]. Thus, an alternative ligand mechanism for Aβ42O is proposed [24]. The metastability and heterogeneity of Aβ42Os make their mechanisms diverse and complex.

On the other hand, it is difficult for larger Aβ42 aggregates to approach neural cell membranes or receptors, but at the same time they are easily deposited in the matrix outside neural cells. The seeding of Aβ42 aggregates (either soluble or deposited form) to the extracellular space is likely to affect the interactions between cells and acellular components in the extracellular matrix (ECM) or between cells, gradually anchors neural cells and makes them become inert, eventually leading to neural cell damage and loss [24]. The recent report [24] also proposes that extracellular Aβ42 aggregates exert detrimental anchoring effects on neural cells, which are significantly attenuated by the application of anti-oligomeric Aβ42 scFv antibodies.

According to a recent report [24], extracellular Aβ42 aggregates (either in soluble/suspended or deposited/attached forms) act as extracellular tethering matrices for neural cells through their anchoring effects on neural cells, thereby gradually tethering the neural cells. It can be speculated that to break free from the “shackles” of extracellular Aβ42 aggregates, neural cells are bound to undergo cascading changes, such as changes in intracellular regulatory substances (in terms of both expression levels and subcellular distribution) or cell behavior (e.g., migration and adhesion) or morphogenesis. Consequently, these stress-induced changes likely disrupt metabolic homeostasis and/or energy balance within the cell. During stress, neural cells that fail to overcome the anchors of Aβ42 aggregates eventually die due to dysfunction and/or energy depletion. This speculation underscores the tethering (or anchoring) role of extracellular Aβ42 aggregates and their hindrance or disruption of neuronal interactions with the normal ECM. The actual situation in the brain may be more serious because intracellular Aβ42 aggregates can damage the anchored or tethered neural cells and accelerate neural cell apoptosis.

However, when the conformational epitopes on Aβ42 aggregates targeted by anti-oligomeric Aβ42 antibodies are equivalent to the toxic sites (i.e., the sites where extracellular Aβ42 aggregates interact with neural cells) on the Aβ42 aggregates, the antibodies can promote the dissociation of these toxic Aβ42 aggregates from target cells through competitive induction, helping neural cells discard these harmful anchors. Such antibodies in all likelihood significantly inhibit/neutralize the neurotoxicity of Aβ42 aggregates and exert neuroprotective efficacies. Inevitably, the development of such effective anti-oligomeric Aβ42 antibodies needs to be based on the results of relevant basic researches. In general, small anti-oligomeric Aβ42 antibodies, such as small anti-oligomeric Aβ42 scFv antibodies, efficiently strip the anchors of Aβ42 aggregates from target cells by facilitating access to the toxic sites where extracellular Aβ42 aggregates interact with neural cells.

In conclusion, analyzing the effects of these antibodies, especially scFvs, on Aβ42O has advanced our understanding of the complex conformations underlying Aβ42O neurotoxicity and has contributed to the development of more desirable anti-oligomeric Aβ42 antibodies. Advances in this field will facilitate the development of novel antibody fragments with superior selectivity and efficacy and, hopefully, good clinical outcomes.

## Figures and Tables

**Table 1:** Conformation-sensitive scFv antibodies mainly targeting oligomeric Aβ species.

Antibody	Binding Sites	Target	Reference
ScFv-h3D6	not reported	Aβ42 monomers, oligomers, and fibrils	[64,65]
NUsc1	not reported	Aβ42 oligomers	[66]
11A5	not reported	Aβ42 oligomers (34 kDa)	[67]
ScFv59	not reported	Aβ42 oligomers and amyloid plaques	[68,69]
A4	not reported	Aβ42 oligomers	[70]
AS	Aβ1–15, Aβ20–33 (by molecular docking)	Aβ42 oligomers and protofibrils (25–55 kDa)	[71]
MO6	Aβ3–4, Aβ15–42 by molecular docking)	Aβ42 oligomers and immature fibrils (18–37 kDa)	[72]
HT6	Aβ1–14, Aβ21–30 (by molecular docking)	Aβ42 oligomers and immature fibrils (18–45 kDa)	[73]
HT7	Aβ1–21/26 (by molecular docking)	Aβ42 oligomers and immature fibrils (23–55 kDa)	[74]

## Data Availability

Not applicable.

## Abbreviations

AD	Alzheimer’s disease
Aβ42	amyloid β-protein (1-42)
APP	amyloid precursor protein
Aβ42M/Aβ42O/Aβ42P/Aβ42F	Aβ42 monomer/oligomer/protofibril/fibril
scFv	single-chain variable fragment
VH or VL	heavy or light chain variable domain
ECM	extracellular matrix
